# Comparison of Rifabutin-Based Versus Rifampin-Based Regimens for the Treatment of *Mycobacterium avium* Complex: A meta-Analysis Study

**DOI:** 10.3389/fphar.2021.693369

**Published:** 2021-09-07

**Authors:** Bahareh Hajikhani, Mohammad Javad Nasiri, Brian C. Adkinson, Taher Azimi, Farima Khalili, Mehdi Goudarzi, Masoud Dadashi, Mukunthan Murthi, Mehdi Mirsaeidi

**Affiliations:** ^1^ Department of Microbiology, School of Medicine, Shahid Beheshti University of Medical Sciences, Tehran, Iran; ^2^ Department of Pulmonary and Critical Care University of Miami Miller School of Medicine Miami, Miami, FL, United States; ^3^ Department of Pathobiology, School of Public Health, Tehran University of Medical Sciences, Tehran, Iran; ^4^ Department of Microbiology, School of Medicine, Alborz University of Medical Sciences, Karaj, Iran; ^5^ Non-Communicable Diseases Research Center, Alborz University of Medical Sciences,Karaj, Iran

**Keywords:** *Mycobacterium avium* complex, rifabutin, rifampin, meta-analysis, systematic rewiew

## Abstract

**Background:** The incidence of Mycobacterium avium complex (MAC) increases as immunosuppressed conditions become more common. MAC's standard treatment regimen includes a macrolide, ethambutol, and a rifamycin, among which rifampin and rifabutin are the most commonly used. Although current guidelines recommend initial therapy for MAC with rifampin, it has been theorized to be less efficacious than rifabutin.

**Methods:** We reviewed the relevant scientific literature published up to February 18, 2020. Statistical analyses were performed with Comprehensive Meta-Analysis Software Version 2.0 (Biostat, Englewood, NJ). The pooled frequency with 95% confidence intervals (CI) was assessed using a random-effect model. We considered P <0.05 as statistically significant for publication bias.

**Results:** After reviewing 3665 records, we identified 24 studies that satisfied the inclusion criteria. Among these studies, 8 had rifabutin in their regimens (rifabutin group) and 16 had rifampin in their regimens (rifampin group). The estimated pooled treatment success rate was found to be 54.7% (95% CI 41.0-67.0%) in rifabutin groups and 67.5% (95% CI 55.7-77.4%) in rifampin groups. There was no evidence of publication bias among the included studies (Egger’s test p-value was 0.7).

**Conclusion:** In this study, it was shown that in comparison to Rifabutin, rifampin has similar treatment success rates in treating MAC. In order to determine the exact preference of each of these drugs, double-blind clinical trial studies are recommended.

## Introduction

Nontuberculous mycobacteria (NTM) are bacteria in the Mycobacterium genus but exclude *Mycobacterium tuberculosis* complex and *M. leprae* (Tortoli, 2014; Shahraki et al., 2015). There is an increasing interest in NTM disease due to the association of NTM infection with immunocompromised states, such as human immunodeficiency virus (HIV), and underlying lung diseases, such as bronchiectasis, chronic obstructive pulmonary disease, and cystic fibrosis (Mirsaeidi et al., 2014a).

*Mycobacterium avium* complex (MAC) is the most common species isolated worldwide but is associated with treatment failure rates of 18–40% (Nasiri et al., 2020). The standard of the care treatment regimen for MAC consists of a macrolide, ethambutol, and a rifamycin. The most commonly used rifamycins are rifampin and rifabutin. Current guidelines recommend initial therapy for MAC with rifampin. Rifabutin is traditionally reserved for severe systemic or recurrent disease (Kim et al., 2019). Rifampin has been preferred for pulmonary MAC due to the reduced tolerance of rifabutin in the elderly who are more likely to have underlying chronic lung diseases such as bronchiectasis and chronic obstructive pulmonary disease (Griffith et al., 2007). Rifabutin is generally well-tolerated in younger HIV populations that are more likely to have disseminated MAC (Crabol et al., 2016). Thus, rifampin is used for pulmonary MAC and rifabutin for disseminated cases by convention. Rifabutin also has less severe drug-drug interaction, which is paramount for those on antiretroviral therapies (Horne et al., 2011).

Despite these differences, there remains uncertainty if one rifamycin is superior for the treatment of MAC. This has led to a considerable variation in practice. Analyzing observational and controlled trials, we herein report a meta-analysis comparing the treatment success rates of rifampin versus rifabutin for pulmonary and disseminated MAC.

## Methods

### Search Strategy

We searched Pubmed/Medline and Embase for studies published up to February 18, 2020. The search strategies were based on (*Mycobacterium avium* or MAC) and (rifabutin or rifampin). This combination of terms was used for searching article title, abstract, or keywords. In Medline and Embase, the relevant MeSH and Emtree terms were also used, respectively. Only studies written in English were selected. This study was conducted and reported according to the PRISMA guidelines (Moher et al., 2009). The study did not require Institutional Review Board approval.

### Study Selection

The records found through database searching were merged, and the duplicates were removed using EndNote X7 (Thomson Reuters, New York, NY, United States). Two reviewers independently screened the records by title, abstract, and full-text to exclude those not related to the current study. Included studies met the following inclusion criteria: 1) patients were diagnosed with MAC using the criteria suggested by ATS/IDSA (Griffith et al., 2007); 2) all study patients were treated with rifampin or rifabutin-containing regimens; and 3) the treatment outcomes were addressed. We defined treatment success as the achievement of culture conversion and completion of the planned treatment without relapse while on treatment. Studies with insufficient information about treatment outcomes were excluded. Conference abstracts, editorials, and reviews were also excluded.

### Data Extraction and Quality Assessment

Two reviewers designed a data extraction form. These reviewers extracted the data from all eligible studies, and differences were resolved by consensus. The following data were extracted: first author name; year of publication; study duration, type of study (RCTS, cohorts, etc.), country/ies where the study was conducted; the number of patients with MAC; age; HIV/AIDS status; treatment protocols (treatment regimens, and duration of treatment), and treatment outcome. The methodological quality of the eligible studies was assessed according to the Cochrane-based criteria (Higgins et al., 2019).

### Data Analysis

Statistical analyses were performed with Comprehensive Meta-Analysis Software Version 2.0 (Biostat, Englewood, NJ). The pooled success treatment rate with 95% confidence intervals (CI) was assessed using a random-effect model. Since prevalence would be affected by the spectrum of populations included, we expected to find significant heterogeneity across the studies. Thus, an a priori decision was made to select the random-effects model because this would give more consistent estimates. The between-study heterogeneity was assessed by Cochran’s Q and the I2 statistic. I2 values of 25, 50, and 75% were considered to represent low, moderate, and high heterogeneity, respectively (Higgins and Thompson, 2002). To minimize heterogeneity, subgroup analyses stratified by study design, disease type, number of drugs used, and treatment length were performed. Publication bias was assessed statistically using Egger’s test (*p* < 0.05 was considered indicative of statistically significant publication bias).

## Results

### Study Selection

**FIGURE 1:**
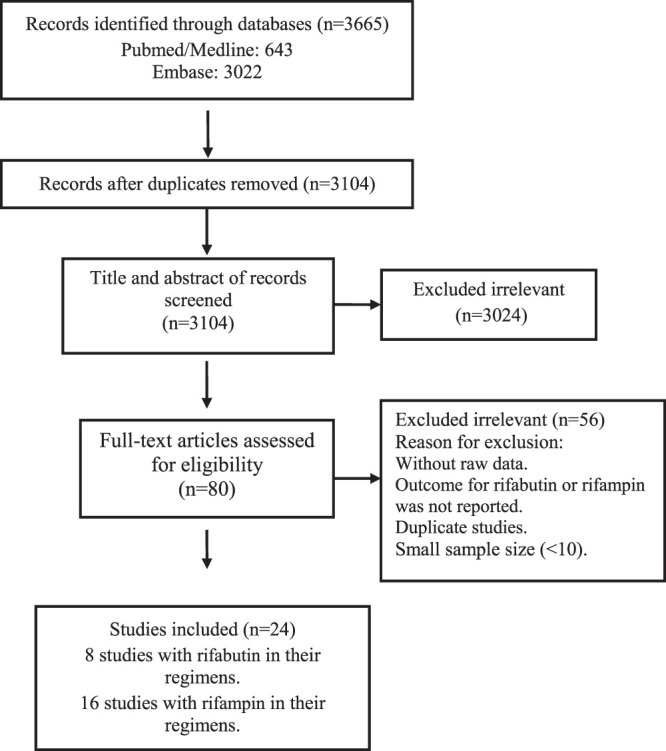
Flow chart of study selection for inclusion in the systematic review and meta-analysis.

The studies included and excluded through the review process are summarized in Figure 1. A total of 3,665 records were found in the initial search; after removing duplicate articles, the titles and abstracts of 3,104 references were screened. Of these, 80 articles were selected for a full-text review. After the full-text review, 24 studies that described the treatment outcomes of rifabutin vs rifampin-containing regimens were chosen for the meta-analysis.

### Characteristics of Included Studies

The characteristics of the included studies are described in Table 1. The study period ranged from 1992 to 2017. The 24 studies comprised 12 RCTs, ten retrospective chart review studies, and two retrospective cohort studies. Eight studies were conducted in Japan, 7 in the United States, 3 in the Republic of Korea, 2 in France, 1 in Canada, 1 in the United Kingdom, 1 in Australia, and 1 in Switzerland. The mean age of the patients ranged from 35 to 71 years. Eight studies used rifabutin in their regimens (rifabutin group), and 16 studies used rifampin in their regimens (rifampin group). The median duration of treatment ranged from 2 to 24 months. Studies sample size range from 18 to 204, with a total number of 1,576 patients. All studies used the definition of treatment success suggested by the ATS/IDSA.

**TABLE 1:** Characteristics of included studies.

Groups	First author	Country and published year	Type of study	HIV prevalence (%)	Mean age	MAC disease	Sample size	Treatment regimens	Median length of treatment (months)	Definition of cure
Rifabutin group	Jo Jo et al. (2014)	South Korea, 2016	Retrospective chart review	0	59	MAC pulmonary disease	51	RFB + CFZ + MXF	5	Culture conversion
	Benson Benson et al. (2003)	United States, 2003	Randomized trial	100	35	Disseminated MAC disease	50	RFB + CLR	4	Culture conversion
							57	RFB + CLR + EMB	4	Culture conversion
	Griffith Griffith et al. (2000)	United States, 2000	Randomized trial	0	63	MAC pulmonary disease	29	RFB + CLR + EMB	6	Culture conversion
	Haefner Haefner et al. (1999)	Switzerland, 1999	Randomized trial	100	40	Disseminated MAC disease	23	RFB + CLR + CFZ	4	Culture conversion
	Cohn Cohn et al. (1999)	United States, 1999	Randomized trial	100	38	Disseminated MAC disease	27	RFB + CLR + EMB	2	Culture conversion
	Gordin Gordin et al. (1999)	United States, 1999	Randomized trial	100	36	Disseminated MAC disease	70	RFB + CLR + EMB	4	Culture conversion
	Shafran Shafran et al. (1996)	Canada, 1996	Randomized trial	100	38	Disseminated MAC disease	97	RFB + EMB + CLR	3	Culture conversion
							90	RFB + EMB + CFZ + CPX	3	Culture conversion
	Dautzenberg Dautzenberg et al. (1996)	France, 1996	Randomized trial	100	25–44	Disseminated MAC disease	55	RFB + CFZ + EMB + INH	3	Culture conversion
Rifampin group	Asakura Asakura et al. (2019)	Japan, 2019	Retrospective chart review	0	68	Refractory MAC pulmonary disease	31	RFP + STFX + CLR + EMB	12	Culture conversion
	Cadelis Cadelis et al. (2017)	France, 2017	Retrospective chart review	17	50	MAC pulmonary disease	34	RFP + CLR + EMB	8	Culture conversion
	Kadota Kadota et al. (2017)	Japan, 2017	Retrospective chart review	NR	66	MAC pulmonary disease	201	RFP + CLR + EMB	18	Culture conversion
	Park Park et al. (2017)	South Korea, 2017	Retrospective chart review	50	62.8	MAC pulmonary disease	204	RFP + CLR + EMB	23	Culture conversion
	Ellender Ellender et al. (2016)	Australia, 2016	Retrospective cohort	NR	61	MAC pulmonary disease	31	RFP + CLR + EMB + AMK	NR	Culture conversion
	Fujita Fujita et al. (2016)	Japan, 2016	Retrospective chart review	NR	66.6	MAC pulmonary disease	18	RFP + CLR + EMB + STFX	12	Culture conversion
	Shimomura Shimomura et al. (2015)	Japan, 2015	Retrospective cohort	NR	71	MAC pulmonary disease	44	RFP + CLR + EMB	12	Culture conversion
	Ito Ito et al. (2014)	Japan, 2014	Retrospective chart review	0	61	MAC pulmonary disease	72	RFP + CLR + EMB	12	Culture conversion
	Miwa Miwa et al. (2014)	Japan, 2013	Randomized trial	0	68	MAC pulmonary disease	32	RFP + CLR + EMB	12	Culture conversion
	Kim Kim et al. (2011)	South Korea, 2011	Retrospective chart review	NR	65	MAC pulmonary disease	21	RFP + CLR + EMB	18	Culture conversion
	Jenkins Jenkins et al. (2008)	United Kingdom, 2008	Randomized trial	0	67	MAC pulmonary disease	66	RFP + CLR + EMB	24	Culture conversion
	Kobashi Kobashi et al. (2007)	Japan, 2007	Randomized trial	0	63	MAC pulmonary disease	73	RFP + CLR + EMB	24	Culture conversion
	Lam Lam et al. (2006)	United States, 2006	Randomized trial	0	60	MAC pulmonary disease	91	RFP + CLR + EMB	12	Culture conversion
	Tanaka Tanaka et al. (1999)	Japan, 1999	Retrospective chart review	0	60	MAC pulmonary disease	39	RFP + CLR + EMB + KAN + OFX	6	Culture conversion
	Wallace Wallace et al. (1996)	United States, 1996	Retrospective chart review	0	60	MAC pulmonary disease	39	RFP + CLR + EMB	6	Culture conversion
	Kemper Kemper et al. (1992)	United States, 1992	Randomized trial	100	35	Disseminated MAC disease	31	RFP + EMB + CFZ + CPX + AMK	3	Culture conversion

EMB, ethambutol; RFP, Rifampicin; RFB, Rifabutin; INH, isoniazid; STM, streptomycin; CFZ, clofazimine; CPX, ciprofloxacin; CLR, clarithromycin; AZM, azithromycin; AMK, amikacin; Mino, minocycline; FQ, fluoroquinolone.

### Quality Assessment

Based on the Cochrane-based tool (Table 2), the included studies had a low risk of bias. The RCTs had a low risk of bias in random sequence generation, incomplete outcome data, and selective reporting. However, blinding of outcome assessment was only fulfilled in 2 studies, and blinding of the participants and study personnel were not reported in 1 of them (Higgins and Thompson, 2002). All except two studies report the treatment outcome, and all of them include detailed follow-up data after treatment.

**TABLE 2:** Assessment of study quality.

Groups	First author	Sampling methods	Blinded	Cross sectional design	Prospective	Incomplete outcome data addressed
Rifabutin group	Jo	Consecutive	No	Yes	No	No
	Benson	Randomized	No	Yes	Yes	No
	Griffith	Randomized	No	Yes	Yes	No
	Haefner	Consecutive	NR	Yes	Yes	No
	Cohn	Randomized	No	Yes	Yes	Yes
	Gordin	Randomized	No	Yes	Yes	No
	Shafran	Randomized	No	Yes	Yes	No
	Dautzenberg	Randomized	Yes	Yes	Yes	No
Rifampin group	Asakura	Consecutive	No	Yes	No	No
	Cadelis	Consecutive	No	Yes	No	No
	Kadota	Consecutive	No	Yes	No	No
	Park	Consecutive	No	Yes	No	No
	Ellender	Consecutive	No	Yes	No	No
	Fujita	Consecutive	No	Yes	No	No
	Shimomura	Consecutive	No	Yes	No	No
	Ito	Consecutive	No	Yes	No	No
	Miwa	Randomized	No	Yes	Yes	No
	Kim	Consecutive	No	Yes	No	No
	Jenkins	Randomized	No	Yes	Yes	Yes
	Kobashi	Randomized	Yes	Yes	Yes	No
	Lam	Randomized	Yes	Yes	Yes	No
	Tanaka	Consecutive	No	No	No	No
	Wallace	Consecutive	No	Yes	No	No
	Kemper	Randomized	No	No	Yes	No

### Treatment Success

The treatment outcomes of 1,576 patients from 24 studies were assessed, and all patients met the criteria for treatment success. A total of 549 patients were identified for evaluating rifabutin-based regimens, and 1,027 patients were identified for evaluation of rifampin-based regimens.

**FIGURE 2:**
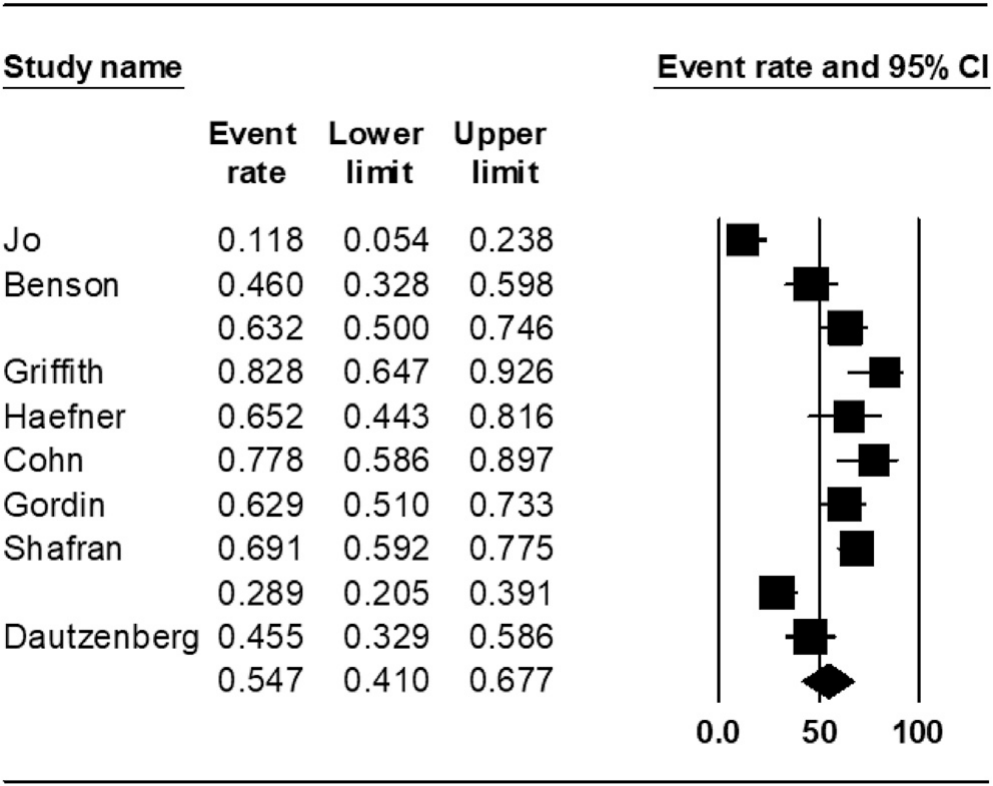
Treatment success rate in rifabutin group.

The pooled treatment success rate was found to be 54.7% [95% CI 41.0–67.0%] in rifabutin-groups (Figure 2). The heterogeneity of the effect estimate (I2) was 88% of the variance, and the *p*-value (Cochran Q test) was <0.001. There was no evidence of publication bias (Egger’s test *p*-value was 0.7).

**FIGURE 3:**
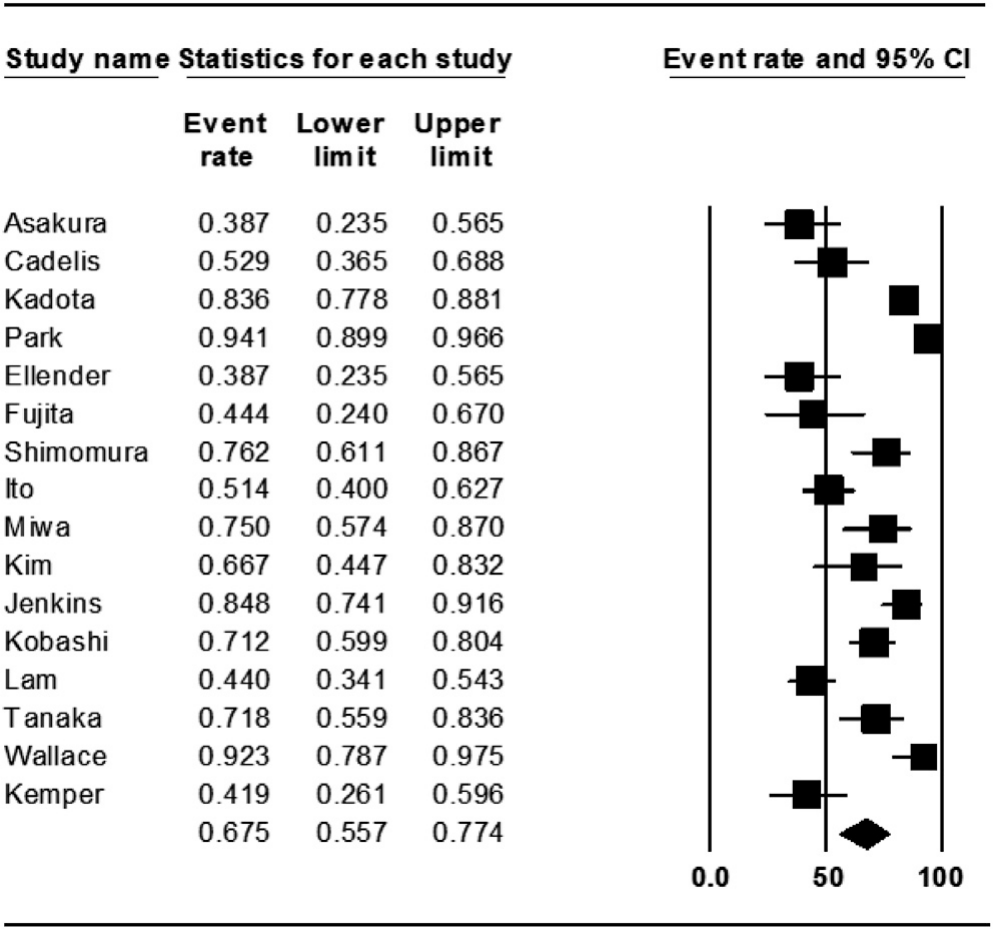
Treatment success rate in rifampin group.

The treatment outcomes of rifampin-containing regimens from 16 studies were also assessed, and the weighted proportion of treatment success among included patients was 67.5% (95% CI 55.7–77.4%). The heterogeneity of the effect estimate (I2) was 90% of the variance, and the *p*-value (Cochran Q test) was <0.001 (Figure 3). There was no evidence of publication bias (Egger’s test *p*-value was 0.7).

### Subgroup Analysis

The subgroup analysis based on treatment regimens is shown in Table3. In the subgroup analyses within the rifabutin-containing regimens, patients’ treatment success rate from the RCTs was higher than that of the other observational studies. The treatment success rate was 60.0% (95% CI, 50.0–72.7) when ≤3 drugs were used, and 36.5% (95% CI, 22.0–53.7) when >3 drugs were used. The treatment success rate for the patients with the pulmonary disease was 44.3% (95% CI, 23.0–90.0), while that with the disseminated disease was 56.8% (95% CI, 44.7–68.2). The result of the Egger test showed no evidence of publication bias (*p*-value = 0.8).

**TABLE 3:** Pooled treatment success rate among subgroups of studies.

Groups	Subgroup, by analysis		Success rate % (95%CI)	Heterogeneity test	
				I^2^ (%)	*p* value
Rifabutin group	Study design	Randomized controlled trials	59.7 (47.6–70.7)	84	0.00
		Retrospective studies	11.8 (5.4–23.8)	0.00	1.00
	Disease type	Pulmonary disease	44.3 (23.0–90.0)	96	0.00
		Disseminated disease	56.8 (44.7–68.2)	83	0.00
	Number of drugs used	≤3	60.0 (50.0–72.7)	85	0.00
		>3	36.5 (22.0–53.7)	75	0.04
	Length of treatment	<12 Months	56.2 (41.2–70.2)	85	0.00
		≥12 Months	48.8 (15.2–83.5)	96	0.00
Rifampin group	Study design	Randomized controlled trials	65.0 (56.0–80.0)	88	0.00
		Retrospective studies	68.6 (53.1–81.0)	91	0.00
	Disease type	Pulmonary disease	69.0 (57.0–79.0)	90	0.00
		Disseminated disease	42.0 (26.0–60.0)	0.00	1.00
	Number of drugs used	≤3	70.0 (54.6–82.0)	92	0.00
		>3	62.2 (44.2–77.5)	82	0.00
	Length of treatment	<12 Months	65.0 (41.7–82.7)	90	0.00
		≥12 Months	70.8 (56.0–82.0)	90	0.00

## Discussion

This study suggests that rifampin is not inferior to rifabutin and may lead to better treatment success rates for MAC. However, there was a significant variation in treatment success rates. The treatment success rate for rifampin was 64.2% (95% CI 55.1–73.3%) compared to 55.2% (95% CI 44.4–66.1%) for rifabutin.

Rifampin has been theorized to be less efficacious than rifabutin due to its effect on the metabolism of other antibiotics (Mirsaeidi et al., 2014b). A study in 1996 found a rifabutin regimen was superior to a rifampin regimen for MAC bacteremia (Shafran et al., 1996). Rifampin is a more potent inducer for the cytochrome (CYP) enzyme system (i.e., strong inducer for CYP3A4 and CYP2C19; moderate inducer for CYP2B6, CYP2C8, and CYP2C9; weak inducer for CYP1A2) while rifabutin only induces CYP3A4 and to a lesser extent (Shulha et al., 2019). Tuloup and colleagues indicated that rifabutin, contrary to rifampin, does not appear likely to cause severe drug-drug interactions, even with sensitive CYP substrates (Tuloup et al., 2021).

Rifampin has been shown to decrease peak serum concentrations of key antibiotics often used in MAC treatment, including clarithromycin, azithromycin, and moxifloxacin (van Ingen et al., 2012). Boorgula et al. in their study found that rifampin monotherapy failed after only 4 days of treatment, and by day 26 of the trial, all MAC population were resistant to rifampin (Boorgula et al., 2021).

Our study results indicate that the pharmacokinetic effect of rifampin is clinically overstated and does not lead to less efficacy compared to rifabutin for the treatment of MAC.

According to our findings, rifampin may be superior to rifabutin for MAC treatment. One explanation for our results is the increased age of patients with human immunodeficiency virus (HIV) in recent decades. Rifabutin has traditionally been recommended for disseminated cases mostly seen in HIV patients, as they were younger with increased ability to tolerate the medication and less potential for interference with CYP enzymes while on antiretroviral therapies (Cowman et al., 2019; Currier and Gandhi, 2021). 22 of 24 studies included in our analysis were published within the last 20 years capturing the present population of aging patients with HIV. Rifabutin is known to have decreased tolerability in these older populations (Finch et al., 2002). With the advent of newer antiretroviral therapies, patients with HIV have experienced increased longevity (Life expectancy of indivi, 2008). As a result, rifabutin regimens are being used in aging populations with decreased tolerability leading to truncation of therapy and treatment failure in MAC. This phenomenon was observed in our study in the shorter duration of rifabutin treatment in the HIV groups.

Limitations of this study are attributable to the potential for confounding variables. The other drugs in the treatment regimen, such as macrolide, can affect as confounders on the treatment’s success rate; however, there was not enough data to separately analyze and discuss the effect of other drugs. Therefore, further investigation such as large, multicenter randomized controlled trials for comparison between Rifampin and Rifabutin success rate is needed to validate the findings. Furthermore, it might be possible to separately discuss the results in HIV and non-HIV subgroups in such trials.

The prevalence of concomitant HIV in the rifampin studies was much less than in the rifabutin studies. HIV was reported in 3 out of 16 studies in the rifampin group (13% of patients) than 6 out of 8 studies in the rifabutin group (85% of patients). One would expect lower treatment success rates in HIV patients due to dysfunctional T lymphocytes, a condition not readily conducive to eradicating MAC. Disseminated MAC is also more prevalent in HIV populations and may increase difficulty with the treatment and the eradication of the bacteria. Other variables center on the lack of consistent dosing protocols and pharmacodynamic parameters among the studies included. Poor treatment success of MAC has been attributed to seldomly met pharmacodynamic indices (van Ingen et al., 2012). The success of rifamycins has been associated with peak concentrations to minimum inhibitory concentration (MIC) ratios, or area under the curve to MIC ratios, which are not reported. Lastly, as half of the included studies were not randomized, there is a risk for type 1 error. Rifabutin may have been employed for more severe cases as is recommended, which would lead to the errant finding that rifampin is a superior treatment (Finch et al., 2002).

The results of this meta-analysis encourage the development of large, randomized controlled trials that can compare the effect of rifampin versus rifabutin in HIV and non-HIV subgroups and disseminated and non-disseminated MAC disease. It should also be noted that double-blind clinical trial studies are necessary to determine the exact preference of each of these drugs.

## Conclusions

This study demonstrates that the treatment success rates of rifampin for MAC are comparable to that of Rifabutin. Our findings also suggest that the pharmacokinetic interactions of rifampin may be overestimated in the clinical setting. Large randomized control trials comparing rifampin versus rifabutin in different patient subgroups are required to analyze further and corroborate these findings. This data can help create a unified clinical practice guideline for MAC.

## Data Availability

The original contributions presented in the study are included in the article/Supplementary Material, further inquiries can be directed to the corresponding authors.
